# *Salmonella enterica* Serovar Agbeni, British Columbia, Canada, 2011

**DOI:** 10.3201/eid1809.120008

**Published:** 2012-09

**Authors:** Marsha Taylor, Shendra Brisdon, Jennifer Jeyes, Jason Stone, Glen Embree, Ana Paccagnella, Linda Hoang, Eleni Galanis

**Affiliations:** British Columbia Centre for Disease Control, Vancouver, British Columbia, Canada (M. Taylor, A. Paccagnella, L. Hoang, E. Galanis);; Fraser Health Authority, Surrey, British Columbia, Canada (S. Brisdon, J. Stone, G. Embree);; Interior Health Authority, Vernon, British Columbia, Canada (J. Jeyes);; and University of British Columbia, Vancouver (L. Hoang, E. Galanis)

**Keywords:** *Salmonella enterica* serovar Agbeni, outbreak, Canada, incubation period, urine, bacteria, foodborne diseases, enteric infections, serovar, Agbeni, salmonella, *Salmonella enterica*

**To the Editor:** Infection with *Salmonella enterica* serovar Agbeni is rare. In Canada, it was reported 8 times during 2000–2010 and never in the province of British Columbia (2011 population 4.5 million) (Public Health Agency of Canada, unpub. data). In June 2011, an outbreak of *S. enterica* ser. Agbeni affecting 8 persons was identified in British Columbia; pulsed-field gel electrophoresis patterns for all isolates were identical. Although no specific source was identified, 2 features were noted: 1) diagnosis through urine specimens for 3 of 8 persons and 2) a longer than typical incubation period for *Salmonella* spp. infection.

In British Columbia, public health authorities interview all reported *Salmonella* spp.–infected persons by using a standard questionnaire (www.bccdc.ca/dis-cond/CDSurveillanceForms) to collect information about potential exposures during the 3 days before date of illness onset. Seven of the ill persons in British Columbia had attended the same wedding on May 14, 2011, which was outside the 3-day period about which they were asked. The person with the earliest reported case (May 16) was not associated with the wedding or with the other ill persons.

We reviewed wedding food sources and preparation. The 7 persons with wedding-associated illness were reinterviewed by using a menu-specific questionnaire; no obvious food source was implicated. The first wedding guest to be reported with enteric symptoms was visiting from outside British Columbia and had assisted with food preparation. In April and May 2011, five persons from the same jurisdiction outside British Columbia in which this wedding guest resided were identified with *S. enterica* ser. Agbeni infection; isolates from these persons had the same pulsed-field gel electrophoresis pattern as those in British Columbia. Also, the ill person who was not associated with the wedding had traveled to that same jurisdiction before onset of symptoms. The original source of infection was probably outside of British Columbia.

Average age of the 8 ill persons was 52.8 years (range 21–82 years). Six were men. One person reported hospital admission. No underlying conditions were documented in any of the 8 ill persons.

Culture results of urine samples were positive for 3 (38%) of the 8 ill persons; feces were not tested. All 3 persons had symptoms of urinary tract infection (UTI), and 2 had fever. All were men and were the oldest persons reported. Two had gastrointestinal (GI) symptoms before UTI symptoms. For 1 person, the interval between onset of GI and UTI symptoms was 15 days.

Approximately 1% of nontyphoidal *Salmonella* spp. infections are detected in urine (*1*,*2*). In British Columbia, ≈3% of *Salmonella* isolates submitted to the reference laboratory are isolated from urine (British Columbia Centre for Disease Control’s Public Health Microbiology and Reference Laboratory, unpub. data). *Salmonella* spp. are more often recovered from urine in adults >60 years of age, children (*2*,*3*), and female patients (*2*,*4*). Immunocompromising conditions and urinary tract structural abnormalities also are risk factors for isolating the organism in urine (*2*,*3*). Also, certain *Salmonella* serogroups or serotypes are more likely than others to be isolated from urine (*2*,*3*). GI symptoms concurrent with or preceding UTI are rare (*4*,*5*). We found no literature to suggest whether *S. enterica* ser. Agbeni is more likely to cause systemic illness or UTI. The only risk factor identified among the persons reported here was older age. Unlike persons in other reports, persons in our report were all men, and 2 reported GI symptoms. The mechanism for UTI in these cases is unclear but could have included ascending and hematogeneous spread.

We calculated incubation periods for GI symptoms for 6 persons as the time between onset of GI symptoms and the May 14 wedding (5 persons) or last travel date (1 person). The incubation period was 5–7 days (average 5.5 days). The incubation period for UTI, which could be calculated for 2 persons, was an average of 25.5 days. Long incubation periods for *Salmonella* spp. infections have been reported (*6*–*9*); reasons include exposure to a low dose of bacteria, specific populations (e.g., young children, child day care attendees), and method of food preparation (*6*–*9*). The age of persons in our investigation did not affect the length of the incubation period. The amount of food eaten was not collected during the interview; however, most persons in our investigation reported eating a wide variety of foods, and 1 reported eating small portions. All food was prepared during the week before the wedding and served cold. This length of time and the potential for temperature abuse could have increased the infectious dose and decreased the incubation period (*6*). In addition, the 1 person with travel-related infection was not exposed to these food items. We found no literature on the incubation period for *S. enterica* ser. Agbeni. The reason for the long incubation period in this investigation is unclear and could be due to host-specific factors, the implicated serotype, or the food source.

The 3-day time frame for exposures was not sufficient to identify appropriate exposures. Expanding the period for collecting exposure information about *Salmonella* spp. infections and the reporting and investigation of persons with *Salmonella* spp. identified in urine to public health authorities might be needed to help identify and solve outbreaks.
